# Improved Bin-Based Basophil Activation Test Facilitates Comparison of Wheat Allergy and Tolerance in Children and Adults

**DOI:** 10.3390/ijms27041620

**Published:** 2026-02-07

**Authors:** Johannes Groffmann, Ines Hoppe, Wail Abbas Ahmed, Dietmar Bast, Sophia Brinster, Seda Altintas, Florian Schusta, Kathleen Weigt, Margitta Worm, Kirsten Beyer, Ria Baumgrass

**Affiliations:** 1 German Rheumatology Research Center (DRFZ), A Leibniz Institute, 10117 Berlin, Germany; 2 Institute of Biochemistry and Biology, Faculty of Science, University of Potsdam, 14476 Potsdam, Germany; 3 Department of Pediatric Respiratory Medicine, Immunology and Critical Care Medicine, Charité—Universitätsmedizin Berlin, 13353 Berlin, Germany; 4 Division of Allergy and Immunology, Department of Dermatology, Venerology and Allergy, Charité—Universitätsmedizin Berlin, 10117 Berlin, Germany

**Keywords:** wheat allergy, wheat tolerance, basophil activation test, flow cytometry, pediatric allergy diagnosis, gliadin, allergy biomarkers

## Abstract

Diagnosing wheat allergy remains challenging due to the use of oral wheat provocations, which implicate risks to patients and highlights the need for safer, non-invasive diagnostic methods. Here, we present the first direct comparison of a pediatric and adult cohort to study wheat allergy and wheat tolerance using an improved and validated basophil activation test (BAT). Blood samples from 24 children and 26 adults, clinically classified as facing oral food challenges, were analyzed using our bin-based BAT, enabling standardized data analysis and visualization. In children, the BAT showed significantly higher median basophil activation in wheat-allergic compared to wheat-tolerant individuals. Receiver operating characteristic analysis revealed that BAT responses to wheat, gluten, and gliadin extracts (area under the curve (AUC): 0.71–0.73) had greater diagnostic accuracies than extract-based wheat and gluten-specific immunoglobulin E (sIgE) measurements (AUC: 0.69, 0.70). However, Tri a 19-sIgE, showed the highest diagnostic performance (AUC: 0.97). In adults, BAT responses did not differ significantly between allergic and tolerant individuals. The bin-based BAT is a robust and reproducible diagnostic tool for wheat allergy diagnosis with automated data analysis capabilities. Significant differences were only evident in the pediatric cohort, indicating age-related immunological differences in basophil responsiveness or immune sensitization profiles. These differences could be linked to immune system maturation, variations in immunoglobulin E (IgE) avidity, or differential expression of the high-affinity IgE receptor (FcεRI) on basophils. While Tri a 19 sIgE was the best single predictor in children, its clinical utility remains controversial due to conflicting results in the scientific literature.

## 1. Introduction

Wheat allergy ranks among the most common food allergies in Europe, with the highest rates observed in children. However, the prevalence varies across diagnostic methodologies [1,2]. Clinically, wheat allergy can manifest with symptoms ranging from mild cutaneous reactions to severe systemic responses, including anaphylaxis or wheat-dependent exercise-induced anaphylaxis (WDEIA) [3], underscoring the need for accurate and reliable diagnostic approaches.

Oral food challenges (OFCs) are the current gold standards for wheat allergy diagnosis, but carry risks of severe allergic reactions and cause patient stress, particularly in pediatric patients. Consequently, recent guidelines such as the one from the European Academy of Allergy & Clinical Immunology (EACCI) emphasize the adjunctive use of indirect tests as skin prick testing (SPT) and the quantification of wheat specific immunoglobulin E (sIgE) to support and refine the diagnostic process. Despite their widespread use, SPT and wheat-sIgE testing show limited diagnostic accuracies for wheat allergy and, in contrast, data on the diagnostic performance of the basophil activation test (BAT) for wheat allergy are missing in these guidelines [4,5,6].

We recently introduced an improved bin-based BAT that incorporates the pattern recognition of immune cells (PRI) approach, enabling automated and standardized data analysis as well as intuitive data visualization [7,8]. BAT is a functional flow cytometry-based ex vivo assay that quantifies allergen-induced activation of basophils. These key effector cells in immunoglobulin E (IgE)-mediated allergic reactions can be activated by IgE- but also non-IgE-mediated activation pathways, giving BATs the potential to complement current diagnostic tools by providing additional mechanistic insight into clinical reactivity [9]. In addition to the BAT, other ex vivo immunoassays, such as the leukocyte adherence inhibition test or lymphocyte proliferation assays, have also been used to investigate immune reactivity to wheat proteins [10,11].

Several studies have tested the utility of the BAT to support the diagnostic procedure for food allergies [12,13]. A small number of studies have explored the application of BAT in wheat allergy and wheat-dependent exercise-induced anaphylaxis. For example, Tokuda et al. showed that a BAT using ω-5 gliadin stimulus achieved ~85% sensitivity and 77% specificity for wheat allergy, outperforming standard sIgE testing [14]. Other studies found that BAT using a subfraction of gliadin called ω-5 gliadin or glutenin could distinguish WDEIA patients from non-sensitized controls. This suggests that BATs using defined wheat components may outperform conventional sIgE testing in identifying wheat-allergic individuals [12]. These preliminary results indicate the potential of the BAT, but these studies were limited to small cohorts, heterogeneous methodologies, a lack of standardized data analysis approaches, and have not been translated into practice. In the present study, we evaluate for the first time the clinical potential of this bin-based BAT in well-characterized cohorts of 24 children and 26 adults with OFC-confirmed wheat allergy and wheat tolerance. By systematically assessing its diagnostic performance, this work aims to address a critical gap in wheat allergy diagnostics and to explore the potential role of BAT as an auxiliary tool in routine clinical practice.

## 2. Results

To evaluate the reliability and diagnostic utility of the BAT for wheat allergy in children and adults, we applied a standardized sample-processing workflow combined with our automated bin-based PRI data analysis approach. This enabled robust identification and quantification of allergen-specific basophil activation. The following section presents data that demonstrates the reproducibility of the assay, its dose-responsiveness, and its diagnostic performance with a focus on pediatric cohorts and comparisons with established sIgE markers. This section also reports on the dynamic ex vivo regulation of the inhibitory receptor FcγRIIB (CD32) on basophils, monitored in a temporally resolved manner during clinically controlled oral food challenges with gluten.

### 2.1. Robust Sample Processing and PRI-Based Analysis Enable Reliable Robust Basophil Activation Measurement

**Figure 1:**
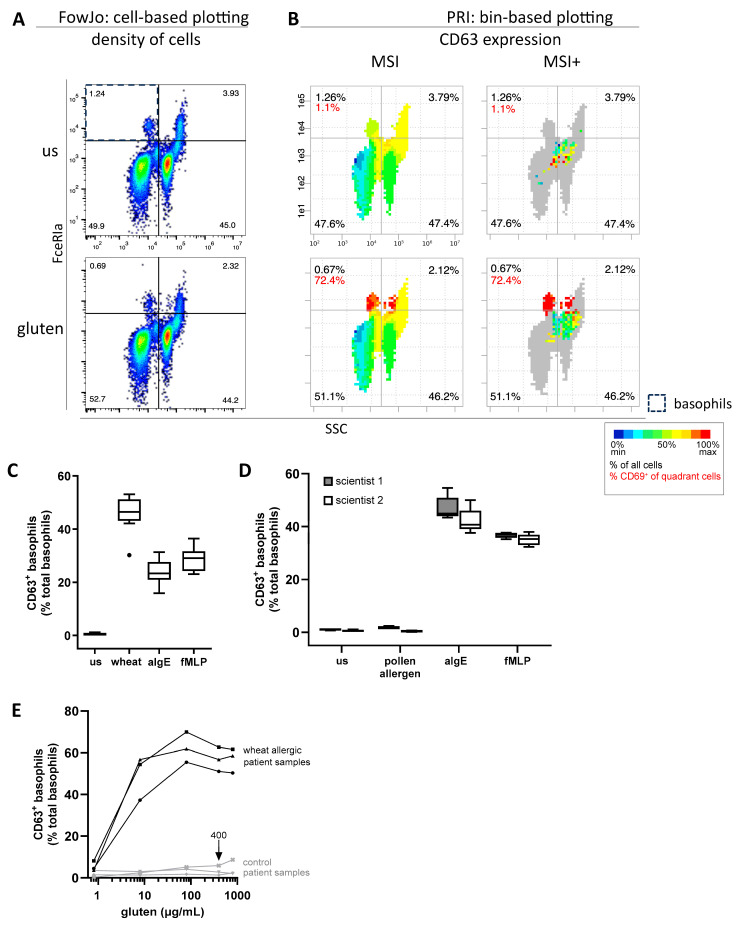
Validation of a bin-based basophil activation test (BAT) for reliable and reproducible detection of basophil activation. (**A**) Flow cytometric single cell-based identification of basophils within erythrolyzed whole blood using side scatter (SSC) and FcεRIα shown as cell density plots. (**Top**): unstimulated sample; (**bottom**): stimulated with 400 µg/mL wheat gluten. Black percentages represent the frequency of all cells per quadrant. Representative samples from a wheat-allergic patient after 15 min incubation at 37 °C. (**B**) Pattern recognition of immune cells (PRI) bin-based 3D visualization of CD63 expression of basophils in the same sample as in (**A**). The activation marker CD63 is shown qualitatively by color-coded intensities as bins (blue = low, red = high) and quantitatively by red percentages representing CD63^+^ frequencies of basophils per quadrant. In mean signal intensity (MSI) plots, bins are based on intensities of all cells; in MSI+ plots, colored bins are based only on intensities of CD63^+^ cells. Black percentages as in (**A**). (**C**) Reproducibility of the BAT demonstrated for 50 µg/mL wheat extract, PBS as negative control (us) and the positive controls 0.25 µg/mL anti-immunoglobulin E (αIgE), and 2.5 µM N-Formyl-Methionyl-Leucyl-Phenylalanine (fMLP). Each condition was measured in technical triplicates based on four independent blood samples from the same wheat-allergic patient (*n* = 12 per condition). Box plot whiskers represent Tukey’s method (1.5 × interquartile range). (**D**) Inter-operator reproducibility tested by two independent scientists analyzing the same blood sample from a ragweed-tolerant control patient in five technical replicates per condition. (**E**) Dose-dependent basophil activation in a wheat-allergic patient in response to increasing concentrations of gluten and minimal or no response observed in non-allergic control individual (technical triplicates). A total of 400 µg/mL gluten was selected as a standard testing concentration.

Reliable basophil identification within erythrolyzed whole blood cells from an OFC-confirmed wheat-allergic patient is shown in Figure 1A. In this two-dimensional single cell-based visualization, each dot represents an individual cell. In contrast, the PRI approach groups single cells into square bins, enabling the three-dimensional visualization of CD63 intensities as a basophil activation marker (Figure 1B). The robust reproducibility of the BAT was confirmed by processing four individual blood samples from an OFC-confirmed wheat-allergic patient in technical triplicates (Figure 1C). The standard deviation (SD) across all 12 measurements for the negative control, wheat allergen, and the positive controls anti-immunoglobulin E (αIgE) and N-Formyl-methionyl-leucyl-phenylalanine (fMLP) was 0.43, 5.96, 4.61 and 4.10, respectively. Further validation was performed by two scientists, individually processing one blood sample from a ragweed pollen-tolerant patient in five replicates per condition (Figure 1D). The SD across all 10 measurements was 0.40 for the negative control, 0.78 for ragweed pollen, 4.74 for αIgE, and 1.73 for fMLP. The BAT also demonstrated dose-dependent quantification capacity, as basophil activation increased proportionally with increasing concentrations of wheat gluten in blood from a representative OFC-confirmed wheat-allergic patient (Figure 1E). These results also highlight 400 µg/mL as an appropriate concentration within the tested range. Assessment of pre-analytical factors showed that basophil stimulation capacity was optimal when blood samples were stored at room temperature and processed within 2 h of collection (Figure S1). These findings demonstrate the reliability of sample processing, ensuring reproducible BAT results and confirming dose-dependent basophil activation in wheat-allergic individuals. Additionally, the PRI approach allows for an advanced standardized data analysis and visualization method, enabling automated BAT analysis as previously described [8].

### 2.2. Wheat-Allergic Children Show Significantly Higher BAT Responses Following Wheat Extract Stimulation than Wheat-Tolerant Children, and Wheat-Allergic Adults Show Significant CD32 Downregulation After Oral Food Challenges with Allergic Reaction Against Gluten

**Figure 2:**
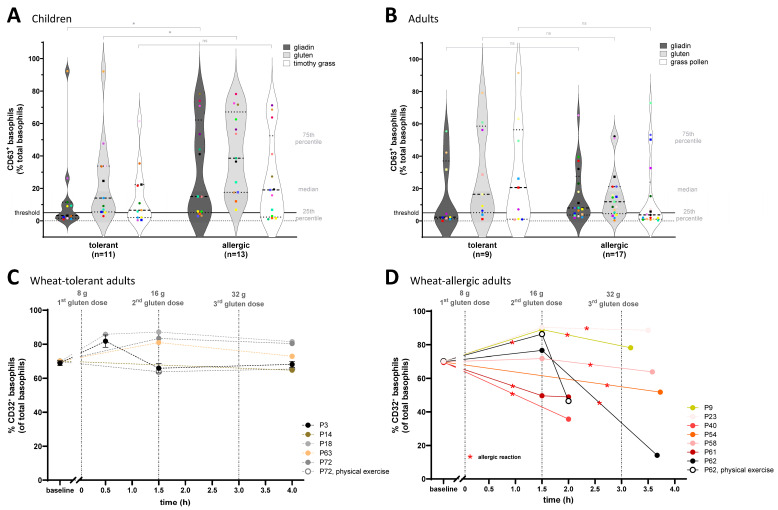
Comparison of basophil activation test (BAT) ex vivo CD63 responses to wheat and pollen extracts between allergic and tolerant individuals in pediatric and adult cohorts and ex vivo monitoring of CD32 on basophils during oral food challenges (OFC) of wheat-allergic and -tolerant adults. Each data point (color and shape) represents an individual patient in the (**A**) pediatric and (**B**) adult cohorts. Basophil activation was measured as the percentage of CD63^+^ basophils relative to the negative control, set to 1%. The horizontal line at 5% indicates the threshold above which basophil responses are defined as positive. Fresh whole blood was stimulated and analyzed by flow cytometry under standardized conditions. Stimulation was performed for 15 min at 37 °C with either wheat gliadin, 300 µg/mL; wheat gluten, 400 µg/mL; or timothy grass pollen extract, 1 µg/mL. (**C**) Each dot represents the quantification of CD32^+^ basophils in unstimulated blood samples collected at multiple time points during clinically supervised OFCs with gluten from wheat-tolerant individuals (*n* = 6, one patient measured in technical triplicates) and (**D**) wheat-allergic individuals (*n* = 8). The label “physical exercise” indicates that the OFC protocol included physical exercise in addition to gluten administration to assess the impact of exercise as an augmentation factor on the allergic response. Data are normalized to baseline levels (set to 70%) for inter-individual comparison. Statistical comparisons are based on a one-sided Wilcoxon rank sum tests; *p* values < 0.05 were considered statistically significant (*) and those ≥0.05 as not significant (ns).

The BAT revealed significantly higher basophil activation in the 13 wheat-allergic children compared to the 11 wheat-tolerant ones after stimulation with gliadin extract and gluten extract (*p* values = 0.027 and 0.041, respectively) (Figure 2A). Median basophil activation to timothy grass pollen was higher in wheat-allergic than in wheat-tolerant children, but the difference was not significant (*p* = 0.126). Additionally, a commercial wheat extract resulted in BAT responses comparable to those obtained with our in-house gluten extract, shown in Figure S2. Noticeably, some allergic patients (light and dark green circles) showed strong basophil activation in response to both wheat allergen extracts but were below the 5% threshold after stimulation with timothy grass pollen extract, suggesting that cross-reactivity between the grain-based wheat extract and pollen-based timothy grass extract does not occur uniformly in all patients, despite the close botanical relationship between the two species. In contrast to the pediatric cohort, wheat-allergic adult patients showed lower and non-significant basophil activation in response to gluten and gliadin (*p* values = 0.156 and 0.110 respectively) and grass pollen extracts (grass pollen = 0.169) compared to wheat-tolerant adults (Figure 2B). These results underscore the potential of our BAT to reveal distinct basophil activation patterns between wheat-allergic and wheat-tolerant children, as well as differences in basophil responses between pediatric and adult cohorts.

To link our cellular findings to clinical responses, we profiled the ex vivo dynamics of the inhibitory receptor CD32 on circulating basophils during standardized gluten oral food challenges. CD32 acts as an important inhibitory checkpoint that can modulate basophil activation, and changes in its expression can reflect regulatory adjustments of immune responsiveness during allergic reactions or allergen immunotherapy [15,16,17]. Serial blood samples were taken at baseline, 1.5 h after the first gluten dose, and 1 h after either symptom onset or completion of uneventful challenges. Our results revealed that CD32 expression on basophils remained stable ex vivo during six gluten oral food challenges in wheat-tolerant patients (Figure 2C). In contrast, in wheat-allergic patients CD32 expression on basophils was significantly reduced (*p* = 0.041) 1 h after experiencing a clinically relevant allergic reaction, with two exceptions out of eight (Figure 2D). These findings suggest that allergic reactions may involve the modulation of inhibitory checkpoints, such as CD32, potentially lowering the activation threshold for basophil degranulation and contributing to symptom development.

### 2.3. BAT Outperforms sIgE to Wheat and Gluten but Tri a 19 sIgE Performs Best in Diagnosing Wheat Allergy in Pediatric Cohort

**Figure 3:**
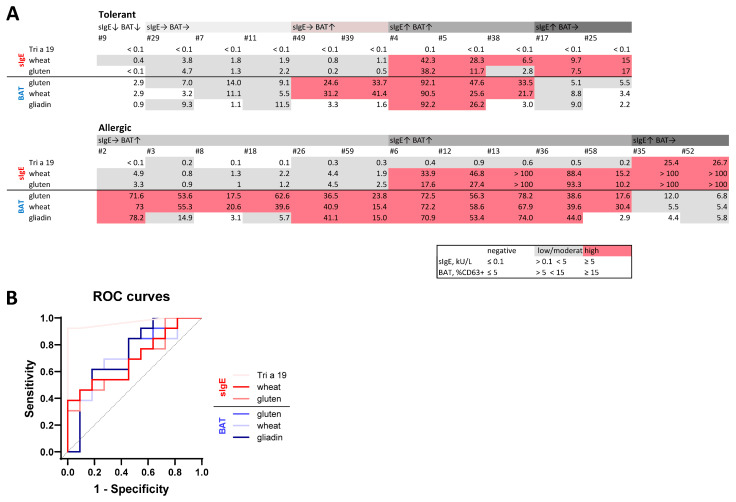
Comparison of BAT and sIgE wheat parameters in the pediatric cohort. (**A**) Classification of BAT and sIgE values from individual patients into categories for wheat-allergic (*n* = 13) and wheat-tolerant (*n* = 11) children. Arrows indicate the relative tendency of sIgE concentrations and BAT responses: ↑ high, → intermediate, and ↓ negative. (**B**) ROC curves for individual BAT and sIgE wheat parameters. Selected statistical parameters are shown in Table 1. The sIgE values < 0.1 kU/L were set to 0.05 kU/L and values > 100 kU/L were set to 101 kU/L for calculations. sIgE was measured using ImmunoCAP (Thermo Fisher Scientific/Phadia, Uppsala, Sweden) with recombinant Tri a 19, wheat (f4), and gluten (f79). BAT was performed using stimulation with 400 µg/mL gluten, 50 µg/mL wheat, or 300 µg/mL gliadin extracts.

For visual and numerical direct comparison of BAT and sIgE between patients and cohorts, values were categorized into negative, low/moderate, and high reactivity classes (Figure 3A). Receiver operating characteristic (ROC) analysis showed that BAT parameters demonstrated good diagnostic performances, with area under the curve (AUC) ROC values higher than those observed for gluten and gliadin sIgE, but lower than for Tri a 19 sIgE (Figure 3B). A detailed comparison of diagnostic performance measures, including sensitivity, specificity, and predictive values, is shown in Table 1. In summary, the presented data demonstrate that the BAT is a robust and reproducible method for detecting wheat allergen-specific basophil responses with low allergen concentrations. Our bin-based analysis delivers reproducible results, enhances visualization, and enables automation of BAT data analysis. Even though the basophil response in wheat-allergic children was significantly higher compared to wheat-tolerant ones, the diagnostic accuracy of Tri a 19 sIgE was the best single marker in ROC-statistics.

## 3. Discussion

This study evaluated the clinical diagnostic performance of an improved, standardized BAT with a bin-based, automated data analysis workflow to compare IgE-mediated (type I) wheat allergy, as defined by current EAACI nomenclature, with wheat tolerance [18]. The assay demonstrated robust reproducibility and required 10-fold lower concentrations of wheat allergens than other published approaches, indicating high sensitivity [12,19,20]. This validated BAT was applied to OFC-characterized pediatric and adult patient cohorts. Consistent with the only other study testing the BAT for wheat allergy diagnosis in children, we first found significantly higher median basophil activation in wheat-allergic children compared to tolerant ones. Second, in the ROC analysis, the BAT outperformed the diagnostic parameter sIgE for wheat extract (f4) [14]. However, the Tri a 19 sIgE included in this study showed the highest diagnostic performance, reflected by the largest AUC of 0.97. Two other pediatric studies comparing sIgEs also found the recombinant Tri a 19 to perform better than wheat extracts [21,22], though the clinical utility of sIgE testing in wheat allergy remains a matter of debate due to inconsistent findings across studies. Potential drivers of these inconsistencies may include geographic and exposure-related differences and variation in clinical phenotypes studied. In particular, studies from Asia for severe phenotypes report a higher utility of Tri a 19 than European cohorts, which typically include broader, milder phenotypes and are affected by pollen-related cross-sensitization [5,23,24,25,26,27,28].

Importantly, this is the first study directly comparing BAT responses to wheat allergens between children and adults. Wheat allergy has been described to behave differently across age groups, for example, in terms of prevalence, progression, and augmentation factors [29,30]. Consistent with these age-related differences, we found no significant differences in BAT responses in adults between wheat-allergic and -tolerant individuals as we did in the pediatric cohort. However, this finding may in part reflect limited statistical power due to the limited sample sizes rather than true age-related differences in basophil responsiveness.

In addition, age-dependent immunological factors, including immune system maturation [31,32], qualitative differences in IgE responses [33], and differential expression of the high-affinity IgE receptor FcεRI on basophils [31,34], have been described and may contribute to the observed findings. In addition thedifferent pre-challenge dietary exposures, with adults on an unrestricted diet and children on a wheat-elimination diet, may have further influenced basophil reactivity. The absence of significant differences in BAT responses to wheat allergens between wheat-allergic and -tolerant adults in our study does not necessarily contradict a previous finding. In the earlier study the authors found significant differences comparing wheat-allergic adults with healthy, non-sensitized controls, whereas we compared allergic adults to clinically tolerant, but wheat-sensitized individuals [12]. Sensitization is the precondition for allergy development. However, the presence of allergen-specific IgE might represent an early or even physiologic immune response rather than a manifest clinical allergy. Moreover, patients are examined at different stages of the allergic inflammatory or tolerance mechanisms, and coexisting allergic conditions, particularly common in adults, can influence basophil responsiveness [35,36]. These factors likely contributed to the smaller differences we observed in the adult cohort.

Finally, we identified a dynamic decrease in basophil CD32 expression ex vivo after allergic reactions, whereas CD32 levels remained stable during uneventful oral food challenges with gluten. Given that basophils express the inhibitory receptor CD32B, whose co-aggregation with FcεRI presumably via allergen–IgG immune complexes can prevent cellular activation, this transient downregulation may reduce inhibitory signaling, enabling degranulation [37]. This study may show the first datamonitoring basophil marker regulation during OFCs ex vivo, as previous studies analyzed changes in granulocyte frequencies but not their activation pattern [38,39]. Given that basophils integrate signals from multiple receptor pathways, changes in surface marker expression reflect complex cellular responses rather than a single IgE-mediated degranulation event. Our findings identify CD32 modulation as a promising biomarker and pharmacodynamic indicator in the context of food allergy.

In conclusion, we present a validated and standardized BAT platform capable of measuring reproducible basophil activation. In children, the test demonstrated good specificity (0.82) but limited sensitivity (0.62), while Tri a 19 sIgE showed the highest diagnostic accuracy among all tested markers. Wheat allergy diagnosis remains challenging due to overlapping symptoms with other wheat-mediated diseases and the absence of reliable standardized biomarkers to differentiate wheat allergy and tolerance [40,41,42]. Further multicenter studies are needed to reduce the number of OFCs in children and adults focusing on methodological harmonization with standardized inter-laboratory protocols, ready-to-use/dry reagents and automated sample processing, combined with automated data analysis. Along with wheat extracts, recombinant proteins as Tri a 19 should also be evaluated to determine the clinical utility of the BAT and to define its role within the routine diagnostic procedure of wheat allergy.

## 4. Materials and Methods

### 4.1. Characterization of Study Population

Patients were grouped based on their response to a double-blind placebo-controlled food challenge (OFC) using increasing amounts of gluten (Kröner Stärke, Ibbenbüren, Germany), or in the case of two children, had a convincing allergic reaction after wheat consumption within the last 6 months. All patients selected for OFC either showed evidence of sensitization to wheat, defined as a positive skin prick test (SPT) or elevated wheat-specific serum IgE levels, or had a previous diagnosis of wheat allergy. Patients with non-IgE-mediated wheat intolerance were excluded from this study. Individuals showing objective allergic symptoms during the OFC, defined according to the Sampson criteria [43], were classified as having an IgE-mediated wheat allergy (wheat allergic). Patients who tolerated the OFC without objective symptoms were classified as wheat tolerant. All patients fasted on the day of the OFC and were asked to avoid large wheat-containing meals on the preceding day. Adults followed a no-elimination diet and maintained an unrestricted diet prior to OFC. In contrast, all children were on a wheat-elimination diet. Clinical history and atopic comorbidities were queried using a questionnaire. Children: A total of 25 children underwent BAT, comprising 14 wheat-allergic and 11 wheat-tolerant patients. One allergic child was identified as an IgE BAT non-responder, defined as a patient with confirmed IgE-mediated allergy who does not show CD63-based basophil activation in the test despite clinical reactivity and had to be removed for further analysis. Detailed pediatric patient characteristics are provided in Table S1. Adults: A total of 27 adults underwent BAT testing, comprising 18 wheat-allergic and 9 wheat-tolerant patients. One allergic adult was classified as IgE BAT non-responder. The clinical characteristics of the adult cohort are summarized in Table S2.

### 4.2. Wheat and Pollen Extracts

Wheat gliadin extract (Gliadin): approximately 50 µg gliadin (Alpha Diagnostics, San Antonio, TX, USA) was mixed with approximately 330 µL 45% (*v*/*v*) PBS in ethanol and approximately 400 mg wheat gluten extract (Gluten) (Kröner Stärke, Ibbenbüren, Germany) was mixed with approximately 1 mL RPMI 1640 Medium, GlutaMAX™ (Thermo Fisher Scientific, Waltham, MA, USA). The allergen mixtures were incubated in a thermomixer at 40 °C, shaking at 1000 rpm for around 1 h. After incubation, the solutions were cooled to RT and centrifuged at 15,000× *g* for 20 min. The protein concentration of the saturated supernatant was measured spectroscopically at 280 nm using a Nanodrop 2000C or by using a Pierce BCA Protein Assay Kit (both Thermo Fisher Scientific). Commercial allergen extracts, including wheat (f4), timothy grass pollen (g6), and sweet vernal grass pollen (g1) (DST, Schwerin, Germany) were reconstituted in double-distilled water according to the manufacturer’s instructions (Table 2).

### 4.3. Basophil Activation Test

The BAT protocol was performed as previously described [7]. Briefly, fresh heparinized whole blood was mixed with RPMI 1640 Medium, GlutaMAX™ (Thermo Fisher Scientific, Waltham, MA, USA) at a 5:2 ratio prewarmed to 37 °C, containing 6.25 μg/mL CD63 VioBlue antibody (clone: H5C6, Miltenyi Biotec, Bergisch Gladbach, Germany) and the respective allergen extract or control in a v-bottom 96-well micro test plate (Sarstedt, Nümbrecht, Germany). Positive controls included 0.25 μg/mL αIgE (Bethyl Laboratories, Montgomery, AL, USA) mimicking the crosslinking of specific IgEs by allergens and 2.5 µM fMLP (Tocris Bioscience, Bristol, UK). PBS was used for the negative control. Stimulation was carried out for 15 min at 37 °C in a water bath and stopped by adding 240 µL BD Phosflow Lyse/Fix Buffer (BD Biosciences, San Jose CA, USA) twice, followed by two washing steps with PBS containing 0.2% bovine serum albumin (BSA). Cells were stained on ice for 20 min using 0.825 µg/mL FcεRIα PE Vio 770 (clone: CRA1, Miltenyi Biotec) as basophil identification marker and 1.25 µg/mL CD32 Alexa Flour 647 (clone: FUN-2, BioLegend, San Diego, CA, USA), which improves automated basophil gating within the PRI-based data analysis. After further washing, the samples were measured on a MACSQuant16 (Miltenyi Biotec, Bergisch Gladbach, Germany) equipped with red (640 nm), blue (488 nm), and violet (405 nm) lasers.

### 4.4. Analysis of Flow Cytometry Data

The PRI approach has previously been described as a method for standardized visualization of flow cytometry raw data, enabling automated identification of basophils and their activation [8]. The essential functionalities of PRI are available under https://github.com/InesHo/PRI-demonstration, accessed on 5 April 2025. Figure 1A was generated using FlowJo™ v10.10 (BD Life Sciences, Ashland, OR, USA).

### 4.5. Statistical Analysis and Data Visualizations

Statistical analyses were performed using R Version 4.4.3 (R Foundation for Statistical Computing, Vienna, Austria) or GraphPad Prism v10.2.2 (GraphPad Software, Boston, MA, USA). Comparisons of qualitative variables between wheat-allergic and wheat-tolerant patients in the supplementary patient characteristics tables were performed using Fisher’s exact test. Continuous variables were analyzed using the unpaired two-sided Wilcoxon test. For Figure 2, statistical comparisons between the tolerant and allergic cohorts were performed using the unpaired one-sided Wilcoxon test.

## Figures and Tables

**Table 1:** A comparison of the diagnostic accuracy of parameters for the basophil activation test and specific immunoglobulin E in 24 children.

	Unit	NPV	PPV	Cutoffs	*p* Wilcoxon		Sensitivity	Specificity	AUC ROC
**sIgE Tri a 19**	U/mL	0.92 (0.65–1.00)	1.00 (0.76–1.00)	0.13	<0.001	**	0.92 (0.67–1.00)	1.00 (0.74–1.00)	0.97
**sIgE wheat**	U/mL	0.58 (0.36–0.77)	1.00 (0.57–1.00)	44.55	0.052	ns	0.38 (0.18–0.64)	1.00 (0.74–1.00)	0.70
**sIgE gluten**	U/mL	0.55 (0.34–0.74)	1.00 (0.51–1.00)	17.30	0.066	ns	0.31 (0.13–0.58)	1.00 (0.74–1.00)	0.69
**BAT-gluten**	%CD63^+^	0.64 (0.39–0.84)	0.80 (0.49–0.96)	35.12	0.041	*	0.62 (0.36–0.82)	0.82 (0.52–0.97)	0.71
**BAT-wheat**	%CD63^+^	0.64 (0.39–0.84)	0.80 (0.49–0.96)	35.41	0.047	*	0.62 (0.36–0.82)	0.82 (0.52–0.97)	0.71
**BAT-gliadin**	%CD63^+^	0.64 (0.39–0.84)	0.80 (0.49–0.98)	13.21	0.027	*	0.62 (0.36–0.82)	0.82 (0.52–0.99)	0.73

Values in parentheses represent 95% confidence intervals. Statistical significance is defined as: * *p* < 0.05, ** *p* < 0.001, ns = not significant. The optimal cutoffs were determined based on the Youden’s J statistic.

**Table 2:** Final allergen concentrations in the basophil activation test.

Allergen	Gluten	Gliadin	Wheat	Timothy Grass
Concentration (µg/mL)	400	300	50	1

## Data Availability

The essential functionalities of PRI are available under https://github.com/InesHo/PRI-demonstration, accessed on 13 August 2025.

## Supplementary Materials

The supporting information can be downloaded at: https://www.mdpi.com/article/10.3390/ijms27041620/s1.

## Abbreviations

The following abbreviations are used in this manuscript:

αIgE	anti-immunoglobulin E
AUC	area under the curve
BAT	basophil activation test
BSA	bovine serum albumin
CI	confidence interval
fMLP	N-Formyl-methionyl-leucyl-phenylalanine
*g*	gravitational force
IgE	immunoglobulin E
MSI	mean signal intensity
MSI+	mean signal intensity of cells above the threshold for the third marker
NPV	negative predictive value
OFC	double-blind placebo-controlled food challenge
PBS	phosphate-buffered saline
PPV	positive predictive value
PRI	pattern recognition of immune cells
ROC	receiver operating characteristic
RT	room temperature
SD	standard deviation
sIgE	specific immunoglobulin E
SPT	skin prick test
SSC	side scatter
WDEIA	wheat-dependent exercise-induced anaphylaxis
