# Supplementary material for: Improved Bin-Based Basophil Activation Test Facilitates Comparison of Wheat Allergy and Tolerance in Children and Adults
Source: Int J Mol Sci. 2026 Feb 7;27(4):1620. doi: 10.3390/ijms27041620 (PMC12940367; doi:10.3390/ijms27041620)
Supplement: Supplementary file 1 [file ijms-27-01620-s001.zip › ijms-3957699-supplementary.pdf]

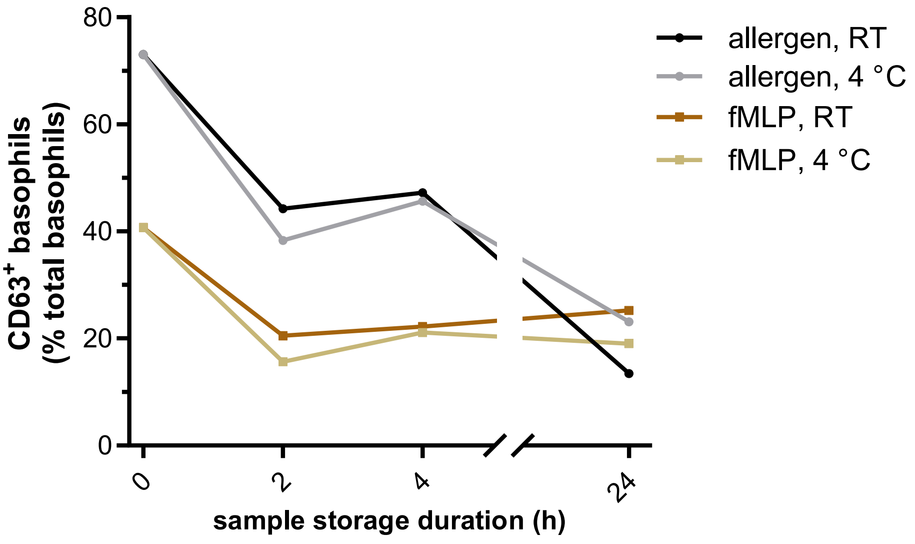

Supplementary Figure S1. Impact of sample storage time and temperature on basophil activation. Basophil activation was evaluated at different time points after blood withdrawal in whole blood stored at room temperature (RT) or 4 °C. Samples were stimulated via two activation pathways with either 1 µg/mL sweet vernal grass pollen extract or 2.5 µM N-Formyl-Methionyl-Leucyl-Phenylalanine (fMLP) in the presence of 2 ng/mL Interleukin-3. Sample processing was performed according to the method section 4.3.

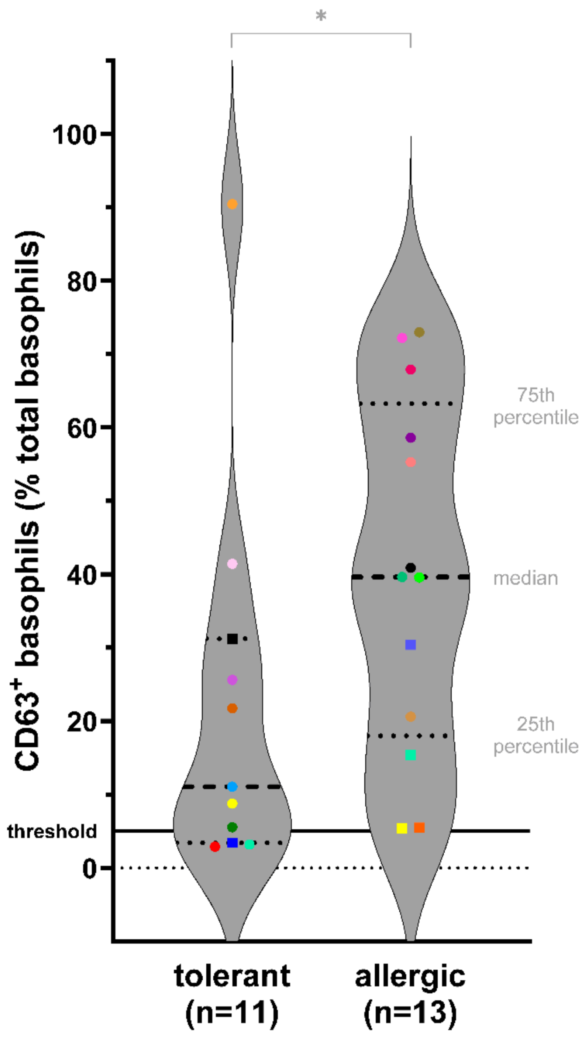

Supplementary Figure S2. **Comparison of basophil activation test responses to wheat extract between allergic and tolerant individuals in pediatric cohorts.** Each data point (color and shape) represents an individual patient. Statistical comparisons are based on a one sided Wilcoxon rank sum test, p values < 0.05 were considered statistically significant (*). Basophil activation was measured as the percentage of $CD63^+$ basophils relative to the negative control, set to 1 %. The horizontal line at 5 % indicates the threshold above which basophil responses are defined as positive. Samples were stimulated with wheat extract, 50 µg/mL. Sample processing was performed according to the method section 4.2.

Supplementary Table S1. Clinical Characteristics of the Pediatric Study Population

	**Total (n = 25)**	**Wheat allergic (n = 14)**	**Wheat tolerant (n = 11)**	***p* value***
Age (y)	5.0 (3.0-6.0)	5.0 (1.5-5.75)	5.0 (3.5-8.0)	0.577
Males (%)	17 (68.0)	9 (64.3)	8 (72.7)	1.0
Asthma (%)	5 (20.0)	3 (21.4)	2 (18.2)	1.0
Allergic Rhinoconjunctivitis (%)	13 (52.0)	5 (45.5)	8 (72.7)	0.111
Eczema (%)	23 (92.0)	12 (85.7)	11 (100.0)	0.487
Other suspected/known food allergy (%)	24 (96.0)	13 (92.9)	11 (100.0)	1.0
AR timothy grass (%)	6 (24.0)	3 (21.4)	3 (27.3)	1.0

Supplementary Table S2. Clinical Characteristics of the Adult Study Population

	**Total (n = 27)**	**Wheat allergic (n = 18)**	**Wheat tolerant (n = 9)**	***p* value***
Age (y)	37.0 (24.5-46.5)	40.5 (32.0-49.2)	25.0 (24.0-34.0)	0.116
Males (%)	13 (48.1)	9 (50.0)	4 (44.4)	1
Asthma (%)	1 (3.7)	1 (5.6)	0 (0.0)	1
Allergic Rhinoconjunctivitis (%)	9 (33.3)	3 (16.7)	6 (66.6)	0.026
Eczema (%)	3 (11.1)	2 (11.1)	1 (11.1)	1
Other suspected/known food allergy (%)	12 (44.4)	7 (38.9)	5 (55.6)	0.448
AR timothy grass (%)	6 (22.2)	1 (5.6)	5 (55.6)	0.008

Data in both tables are presented as median (interquartile range) or number (percentage).

* *p* values refer to the comparison between wheat allergic and wheat tolerant patients using the unpaired two sided Wilcoxon Test for the parameter age and the two tailed Fisher's exact test for all other parameters. A *p* value of < 0.05 was considered statistically significant.
